# Congenital Gerbode Defect: A Left Ventricular to Right Atrial Shunt—State-of-the-Art Review of Its General Data, Diagnostic Modalities, and Treatment Strategies

**DOI:** 10.3390/jcdd11060166

**Published:** 2024-05-28

**Authors:** Lone Winter, Brigitte Strizek, Florian Recker

**Affiliations:** Department of Obstetrics and Prenatal Medicine, University Hospital Bonn, Venusberg Campus 1, 53127 Bonn, Germany; lone.winter@ukbonn.de (L.W.); brigitte.strizek@ukbonn.de (B.S.)

**Keywords:** congenital Gerbode defect, intracardial shunt, diagnostic imaging, echocardiography, defect closure

## Abstract

The congenital Gerbode defect is defined as an abnormal communication between the left ventricle and the right atrium. This review aimed to summarize existing evidence, shed light on the clinical implications, and identify knowledge gaps. The systematic literature search was conducted in the PubMed and Google Scholar medical databases using specifically selected keywords. The inclusion of each publication was assessed according to predefined eligibility criteria based on the PICOM (Population, Phenomenon of Interest, Context, Methodology) schema. Titles and abstracts were screened independently by two authors. Available full-text versions of included publications were reviewed and relevant information was extracted. A total of 78 reports were included. The compilation of all congenital Gerbode defect cases described in the literature revealed a variety of clinical presentations comprising dyspnea, palpitations, growth retardation, and asymptomatology. A suitable multimodal diagnostic approach for newborns consists of auscultation, TTE, and optionally TEE and MRI. Because of its rarity, diversity of findings, unknown pathophysiology, and similarity to more common cardiac diseases, the diagnostic challenge remains significant. To prevent untreated long-term sequelae, early individualized treatment is recommended. Surgical defect closure is preferred to device closure for evidence reasons, although major developments are currently taking place. In conclusion, the congenital Gerbode defect provides a diagnostic challenge for pediatricians to allow early diagnosis and intervention in order to improve patients’ quality of life.

## 1. Introduction

The Gerbode defect is defined as a rare abnormal communication between the left ventricle (LV) and the right atrium (RA). This malformation permits a direct shunting of blood from the LV to the RA, bypassing the normal pathway. This lesion can be either congenital or acquired in nature [1,2,3]. The acquired form is much more common than the congenital form and is described frequently in the literature. Causes of acquired defects include trauma, endocarditis, myocardial infarction or invasive cardiac procedures [4,5,6].

However, this systematic review of the literature focuses on the congenital form of the Gerbode defect. Although congenital LV-to-RA shunt represents a rare disease, accounting for less than 1% of all congenital cardiac defects [1,5,7,8,9,10,11], it has significant clinical importance [12]. Various comorbidities and complications that may occur during the course of disease have a considerable impact on the outcome. This results in a low quality of life and a high morbidity and mortality of patients with an untreated congenital Gerbode defect. Therefore, providing an accurate detection and assessment of the defect is of fundamental importance to ensure early intervention.

Conducting a systematic literature review can offer a comprehensive approach to assess existing research, synthesize evidence, and identify knowledge gaps. This review aims to provide a general overview of the congenital Gerbode defect, as a rare disease and one that is underrepresented in the literature, as well as to highlight clinical presentations, diagnostic challenges, and treatment strategies. The compilation of all cases of congenital Gerbode defects described in the literature (to the best of our knowledge) is intended to contribute to early diagnosis in neonates to improve patient outcomes.

## 2. Methods

**Figure 1:**
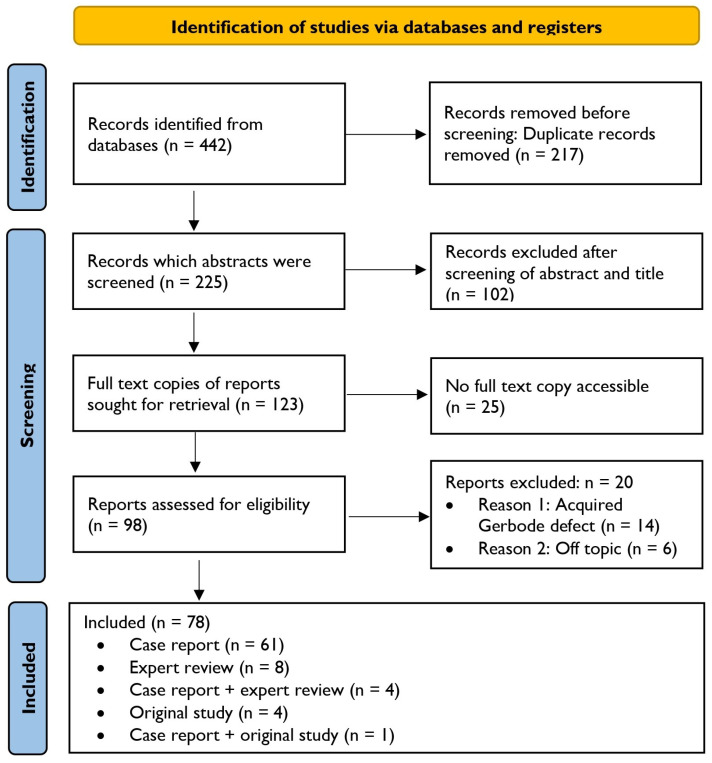
Identification of studies via databases and registers. This flow diagram shows the study selection process according to Preferred Reporting Items for Systematic Reviews and Meta-Analyses (PRISMA) guidelines 2020.

The systematic literature search was performed according to the updated Preferred Reporting Items for Systematic Reviews and Meta-Analyses (PRISMA) Statement for reporting systematic reviews and meta-analyses of studies (see Figure 1) [13,14,15].

The search for literature was initiated in April 2023. The databases PubMed and Google Scholar were searched for relevant publications in English on the condition of Gerbode defect using the keywords [Gerbode defect], [left ventricular to right atrial shunt], and [heart defect] plus [congenital] plus [diagn*] plus [prenatal] or [fetal]. Duplicates resulting from the search in several databases were removed. Titles and abstracts of reports identified from the databases were screened by two authors for fulfillment with the eligibility criteria using a blinded strategy. Eligibility criteria, in terms of target study characteristics, were defined using the PICOM (Population, Phenomenon of Interest, Context, Methodology) schema (see Table 1) [13].

All included reports addressing the clinical characteristics, diagnosis, and treatment strategy of the congenital Gerbode defect were checked for the availability of a full-text version. Fulfilling the criteria described above, 78 reports were conclusively included in the research of data, comprising case reports, expert reviews, and original. While containing essential information on the congenital Gerbode defect, studies primarily focusing on the acquired Gerbode defect were additionally included. A total of 344 studies were excluded due to duplication, noncompliance with eligibility criteria (e.g., off topic or solely focused on acquired Gerbode defect considerations), and inaccessibility of full-text versions. For data and information extraction, the full-text versions of all included sources were read and analyzed twice by two authors (L.W. and F.R.). To reduce the risk of bias in our study and to assess bias in the included studies, the authors worked independently. No automation tools were used. Data pertaining to general information on the congenital Gerbode defect, pathophysiological mechanisms, clinical presentations, diagnostic tools, therapeutic options, and potential complications were systematically extracted. The aspects that structure this article as subheadings were used to guide data extraction. In addition, all case reports of the congenital variant of the Gerbode defect described in the literature were tabulated and compared with respect to general patient characteristics, clinical symptomatology, diagnostic procedures, treatment strategy, and outcome, and subsequently analyzed using descriptive statistical methods (see Table 2). Similarities and differences in diagnostic strategies and treatment options are highlighted. Missing data were handled using complete case analysis and are indicated by n.a. (not available). This review was registered in PROSPERO, the international prospective register of systematic reviews (ID: 535271).

## 3. Results

A total of 78 reports were included in the literature review, and 47 cases of congenital Gerbode defect documented in the case reports are listed in Table 2 and differentiated concerning patient characteristics, symptomatology, diagnostic tools used, therapeutic strategies, and outcomes.

### 3.1. General Information about Gerbode Defect

Thurman et al. were the first to discover the phenomenon of left ventricular to right atrial communication during an autopsy in 1838. In 1857, Buhl also described a case of direct communication between the left ventricle and the right atrium. About one hundred years later, in 1956, Kirby et al. performed the first successful surgical closure of the defect using hypothermia and inflow occlusion technique at the Hospital of the University of Pennsylvania. Subsequently, in 1958, the namesake of the Gerbode defect, the surgeon Frank Gerbode, performed a successful defect closure in a case series of five patients at the Stanford University and described the anomaly as follows: “The lesion consists of a high ventricular septal defect associated with a defect of the septal leaflet of the tricuspid valve which allows left ventricular blood to enter the right atrium [1,26,33,35,43,44,45].

**Figure 2:**
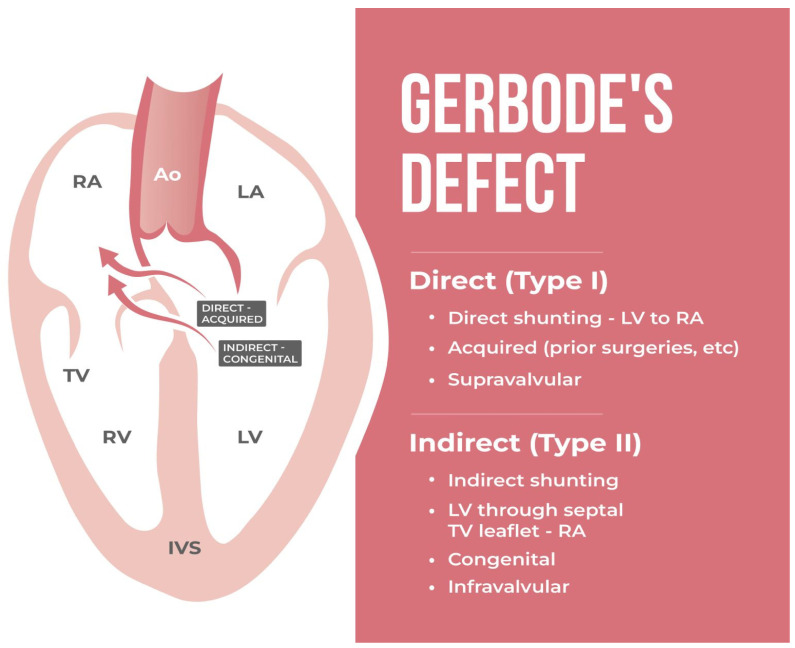
Anatomical classification of the Gerbode defect. The direct and indirect types of the Gerbode defect, according to the anatomical classification by Sakakibara and Kono are shown. Abbr.: LV: left ventricle, RA: right atrium, TV: tricuspid valve.

According to the current anatomical classification by Sakakibara and Kono, three types of Gerbode defects can be differentiated [12,40] (see Figure 2). The classification is based on the localization of the anatomic deficiency of the membranous septum characterizing the Gerbode defect. Depending on the positional relationship to the septal leaflet of the tricuspid valve (TV), the membranous septum is divided into two parts [4]: the interventricular part, which is apically localized, and the atrioventricular part, which is basally localized. Once the TV attaches approximately 1 cm apical to the mitral valve, there exists an atrioventricular part of the septum separating the LV from the RA [46,47,48]. Due to an apical displacement of the tricuspid valve as compared to the mitral valve, the septal leaflet divides the membranous septum into an interventricular and atrioventricular part; thus, the atrioventricular septum separates the left ventricle from the right atrium [44].

The type I form, which is also referred to as direct or supravalvular defect, involves the atrioventricular portion of the membranous septum and is thus localized above the tricuspid leaflet. Herein, it is the “true” Gerbode defect with direct shunting from the left ventricle into the right atrium. According to the STS Congenital Heart Nomenclature and Database Project, the Gerbode defect is defined as type I comprising a direct shunt [43,45].

Type II, also titled indirect or infravalvular defect, affects the interventricular part of the membranous septum, localized below the tricuspid leaflet. Additionally, a tricuspid valve defect is present. Therefore, it represents a perimembranous ventricular septal defect with a shunt originating in the LV and targeting the RA bypassing the defective tricuspid valve. The retrograde flow through the TV may be caused by a cleft, a widened commissural space, a perforation, abnormal chordae, and other deformities.

Type III, the intermediate form, combines the characteristics of types I and II. There is a defect in both the atrioventricular and interventricular portions of the membranous septum [3,33,49].

The incidences in literature of the three types amount to 76%, 16%, and 8%, respectively [12,25,33]. The congenital form of the Gerbode defect mainly comprises types II and III [1,10]. Our case analysis revealed an incidence of 38.3% for type I, an incidence of 48.9% for type II, and an incidence of 12.8% for type III. As a result of our focus on cases of the congenital Gerbode defect, the incidence of type I detected by our review is lower than reported in the literature. Consequently, the incidences of type II and III, which represent the predominant types of the congenital Gerbode defect, are somewhat overrepresented in our case cohort.

### 3.2. Embryology

The embryological development and mechanisms of congenital LV-RA shunt are not fully understood and described, though an endocardial fusion defect is presumed [5,38]. In early embryonic stages, between the 27th and 37th day of development, the septation of the four cardiac cavities is initiated by the approach and fusion of endocardial cushions. The lumen of the formerly undivided cardiac cavity is separated into two canals forming the membranous portion of the interventricular septum, atrioventricular canals, and valves, as well as aortic and pulmonary canals. The Gerbode defect involves the atrioventricular portion of the membranous part of the septum, allowing abnormal shunting between the LV and the RA. Presumably, the defect in this septal portion is caused by endocardial cushion defects and malfusion. Furthermore, the septal leaflet of TV derives from the atrioventricular portion of the membranous septum, which is malformed in the Gerbode defect. This may explain the development of type II and III Gerbode defects, in which retrograde flow through the TV occurs [33].

A multifactorial genesis of the congenital Gerbode defect is presumed, with genetics playing an important role [12]. Genotype-phenotype analysis in patients with the congenital Gerbode defect was performed by Borkar et al. Transcription factors regulating the cardiac embryological septation process were found to be NKX2-5, GATA4, and TBX5 [10]. A correlation of genotypic variants and mutations of these genes with cardiac septal defects is described. Specific etiological mechanisms of the presumed multifactorial genesis of the congenital Gerbode defect have not yet been discovered. Further studies in this regard are needed [2,33].

### 3.3. Hemodynamic Aspects

Due to the anatomic malformation of the atrioventricular septum, abnormal shunting occurs between the LV and the RA. The large systolic pressure gradient between the two cardiac chambers causes a high-velocity systolic jet from the LV into the RA. Blood shunting into the RA, and subsequently into the right ventricle (RV), causes volume overload and increased right ventricular preload, which results in enlargement of the right heart cavities. As the right atrium is in communication with the capacitive vena cava system, its enlargement occurs without significant change in pressure. If not corrected, right atrium strain can lead to right heart failure with systemic venous congestion. In addition, the increased right ventricular preload causes pulmonary circulation overload with a subsequent volume loading and enlargement of the left heart cavities [1,33,38]. Thus, in the case of a significant shunt with therapeutic delay, the acyanotic Gerbode defect [44] may result in dilatation of all cardiac cavities, pulmonary artery hypertension and congestion, as well as low cardiac output and a decline in global cardiac function [11,50].

### 3.4. Clinical Manifestations

The clinical manifestations of the congenital Gerbode defect vary from asymptomatic to severe heart failure and, at worst, death [47]. The extent of symptomatology depends on the size of the defect and the volume and duration of the LV-RA shunt [7,12]. The systematic literature review by Yuan [5] reported that more than 25% of patients suffering from the congenital Gerbode defect were asymptomatic in the context of incidental findings, whereas dyspnea, systolic heart failure, and fever accounted for the prevalent clinical manifestations [50]. Dyspnea is caused by pulmonary arterial hypertension with congestion. Other symptoms described in the literature include feeding difficulties, growth retardation, chest pain, and, in the case of large defects, nonspecific signs of right or left heart failure, such as shortness of breath, peripheral edema, fatigue, and weakness [1,33].

The analysis of all cases of the congenital Gerbode defect detected in our systematic review of the literature revealed the following concerning its clinical manifestations (see Table 2). Of a total of 47 cases, no information on symptomatology was provided for eight cases (17.0%). Of the remaining thirty-nine patients (83.0%), eight patients (20.5%) presented as asymptomatic. The most prevalent clinical manifestations included dyspnea (fourteen cases (35.9%)), palpitations (eight cases (20.5%)), as well as shortness of breath, fatigue, and failure to thrive (six cases each (15.4%)). Other symptoms included chest pain, fever, respiratory distress, sweating, poor appetite, dizziness, peripheral edema, cough, and disturbed sleep.

### 3.5. Diagnostic Modalities and Imaging Techniques

#### 3.5.1. Physical Examination

Cardiac auscultation during physical examination provides characteristic evidence of the presence of an LV-RA shunt. The hallmark finding is a loud, harsh, grade III-IV pansystolic murmur along the left sternal border in the fourth or fifth intercostal space. This heart murmur is consistent with breathing, audible over the entire precordium, radiates posteriorly, and often is associated with a thrill [33,46]. To distinguish from a press jet murmur in a ventricular septal defect (VSD), Vogelpoel described that VSD heart murmurs show a higher and breath-dependent frequency [1]. Differentiation from an atrial septal defect is possible because there is no splitting of the second heart sound in the Gerbode defect. In addition, an atrial septal defect is characterized by a midsystolic injection murmur of variable intensity with punctum maximum above the pulmonary valve, which differs in localization and type from the murmur in an LV-RA shunt [26]. Our analysis of the cases of congenital Gerbode defect described in the literature revealed typical auscultation findings, as described above, in 36 of 39 patients (92.3%; missing data in 8 cases (17.0%)). Just three patients (7.7%) presented without abnormalities at auscultation.

Indicators of right heart failure include increased jugular venous pressure, hepatomegaly, and peripheral edema [1,12].

#### 3.5.2. Twelve-Lead Electrocardiogram

Twelve-lead electrocardiogram findings described by Gerbode that are indicative, but not pathognomonic, of an LV-RA shunt, include a delay in right ventricular activation resulting in an incomplete right bundle branch block. In addition, a prolonged P-R interval and peaked right atrial P waves indicate right atrial dilatation [26]. Atrial fibrillation may also occur [11].

Among the cases of the congenital Gerbode defect tabulated in the context of our review, 32 of 47 patients (68.1%) underwent an electrocardiogram, of which 16 patients (50.0%) showed the abovementioned findings, whereas a sinus rhythm was present in the remaining 16 patients (50.0%). Clinical symptoms were observed in 85.7% of patients with abnormal ECG and in 75.0% of patients with normal ECG. This illustrates the limitations of the ECG as a diagnostic tool. The presence of the above-mentioned typical ECG findings does therefore not correlate with a more severe symptomatology or severity of the defect.

#### 3.5.3. Echocardiography

The characteristic of the Gerbode defect is a systolic high-velocity jet directed from the LV into the RA, with a maximum velocity (Vmax) of >4 m/s. Within the case of an indirect Gerbode defect, the jet is simultaneously directed from both the LV into the RV and from the TV into the RA [25], combined with a high-frequency systolic fluttering of the tricuspid valve, which is easily detectable using M-mode [33]. Frequently, the septal leaflet of the TV completely covers the defect, forming an aneurysm and directing a large turbulent jet into the RA [44]. Yuan summarized various LV-RA shunt manifestations described in case reports: elongated sail-like, valve-like, fistula-like, and cystic thin-walled structures, as well as windsock-like tunnels [5]. A further echocardiographic finding, representing a prerequisite for the high-velocity jet, is a high Doppler gradient between the LV and the RA [44,51]. In the study of Kelle et al., examining a series of six congenital cases of the Gerbode defect, the average pressure gradient was found to be 109 mmHg, with a range from 65 to 150 mmHg [51]. Due to the hemodynamic characteristics of the LV-RA shunt, right atrial dilatation typically results, which is evident on echocardiography. In posterior and anterior views, the enlarged right atrium forms the lower left portion of the cardiac shadow. In addition, there may be an enlargement of the right ventricle and pulmonary artery, along with pulmonary arterial congestion. Depending on the size and relevance of the shunt, left heart involvement and volume overload may be present [12,26]. The Qp/Qs ratio can be used to assess the hemodynamic relevance of the intracardiac shunt, defining a small, restrictive shunt without cardiac chamber enlargement as Qp/Qs < 1.5:1 and a large, significant shunt with cardiac chamber enlargement as Qp/Qs > 2:1 [5].

Silbiger et al. summarized several key echocardiographic clues suggesting a Gerbode defect, including (1) atypical jet direction, (2) persistent shunt flow into dias-tole, (3) lack of ventricular septal flattening, (4) no right ventricular hypertrophy, and (5) normal diastolic pulmonary artery pressure as estimated from the pulmonic regurgitant velocity [52].

Transthoracic echocardiography (TTE) is frequently performed as a first-line imaging modality for the Gerbode defect, representing the gold standard of diagnosis. TTE combined with Doppler interrogation provides a widely available, rapid, and noninvasive diagnostic tool [22] for the assessment of location, size, morphology, hemodynamic relevance, and TV involvement of the perimembranous interventricular septal defect [31]. The diagnostic accuracy amounts to 62.2%, along with 13.4% incon-clusive diagnosis, 9.8% missed diagnosis, and a 14.5% misdiagnosis rate [44].Which The transthoracic echocardiographic axis that is most appropriate in terms of sensitivity for detailed visualization of the high-velocity jet is a matter of controversy among various authors. For instance, Şaylan Çevik preferred the combined use of the parasternal short axis, apical short axis, and subcostal views [7], whereas Colomba et al. rejected short axis projections due to lack of sensitivity [18]. Desai et al. reported that the parasternal long axis, parasternal short axis, and apical four-chamber views can lead to a misdiagnosis of tricuspid regurgitation due to lack of shunt localization [20]. Yuan summarizeded up that the subcostal four-chamber view provides the best visualization of the shunt be-cause no bone or lung tissue obstructs the view of the heart [5]. In addition, combined with a modified parasternal short axis, this axis allows simultaneous visualization of the right atrial and left ventricular outflow tracts. By adjusting the color setting, the accuracy of the TTE can be increased [47]. Using pulsed Doppler mapping, the shunt can be de-tected by generating a high-turbulence audio signal. Continuous Doppler waves might help in detection and measurement of trans-shunt peak systolic velocities. Color flow imaging is most sensitive in terms of localizing defect position and shunt flow [37].

In addition to transthoracic echocardiography, transesophageal echocardiography (TEE) is an elementary mainstay of diagnostics featuring higher preciseness and repre-senting the most sensitive diagnostic modality for assessing the Gerbode defect [11,12,53,54,55]. According to the literature review by Yuan, TEE was superior to TTE in terms of diagnostic accuracy, rate of missed diagnoses, and rate of inclusive diagnoses, whereas misdiagnosis rates did not differ between TTE and TEE [5]. TEE can be used to more accurately visualize the anatomic location and direction of the shunt, as well as to avoid diagnostic errors like a ruptured sinus of Valsalva, VSD, and tricuspid regurgitation [6]. The most suitable views to display the LV-RA shunt are described as the midesophageal four4- chambers view, midesophageal right ventricle inflow–outflow view, and midesophageal reversed four4 -chamber view (130–160°) [50].

As an extension to 2D echocardiography, which is limited in terms of exact ana-tomical localization and relations of the shunt, 3D echocardiography can be performed. It provides a fast, detailed three-dimensional view of the shape and size of the defect, of the localization and course of the shunt, and of tricuspid defects in the case of an indirect Gerbode defect. Taskesen et al. outlined that the most notable advantages of 3D TEE compared to other imaging modalities are its portability and the ability to visualize the defect in a direct “en face” view [46]. In addition, other anatomical structures such as the tricuspid valve leaflet, right ventricular outflow tract, and aortic valve can be spatially visualized. Its accurate assessment facilitates differentiation from misdiagnoses such as ruptured sinus of Valsalva, endocardial cushion defects, VSDs, and tricuspid regurgita-tion [4,33,46,56]. These findings may have clinical implications for decision-making regarding indications for and type of treatment, guiding the sizing and placement of percutaneous closure, as well as and planning surgical management [6,57,58].

In the case reports of congenital Gerbode defects analyzed in our review, a 2D TTE was performed in 42 of 47 patients (89.4%). This was complemented by 3D TTE in four cases (8.5%), by 2D TEE in nine cases (19.2%), and by 3D TEE in one case (2.1%).

#### 3.5.4. Chest X-ray

Chest X-ray findings described in the literature include moderate cardiac enlargement, particularly of the RA, RV, and pulmonary artery, along with increased pulmonary vascularity [25,26,27,44].

In the case reports we analyzed, findings of a chest X-ray were described in 20 of the 47 patients (63.8%), of whom 17 (85.0%) showed pathologic abnormalities and 3 (15.0%) showed normal findings.

#### 3.5.5. Cardiac Catheterization

Cardiac catheterization can be used in particular to quantify the hemodynamic relevance of the intracardiac shunt. A characteristic finding of Gerbode defect is an increase in oxygen saturation between the superior vena cava and the RA [1,32,59]. The presence of a shunt can be confirmed by left ventriculography with contrast opacification of the RA prior to the RV, originating from the LV [7,60]. Despite its diagnostic importance for hemodynamic evaluation and clarification of complex setups, cardiac catheterization has been replaced by noninvasive cardiac imaging techniques [46].

Cardiac catheterization was performed in 13 of 47 patients (27.7%) in the cases of our systematic review.

#### 3.5.6. Magnet Resonance Imaging (MRI)

MRI provides a diagnostic tool for confirming the diagnosis of a septal cardiac de-fect. It offers detailed anatomic information on the size and location of the defect, quantifies the shunt flow, and verifies the measurements of ventricular volumes. How-ever, this modality is expensive, time-consuming, not widely available and or accessible, as well as and requires pharmacologic sedation in young children [7,12,33,46,61,62]. Cardiac MRI was performed within the diagnostic workup in six 6 of the 47 cases (12.8%) documented by us.

#### 3.5.7. Computed Tomography Angiography (CT-A)

CT-A of the chest represents a noninvasive modality to access the cardiac function and cardiac structures with high spatial and temporal resolution [63]. It allows detailed visualization of cardiac shunts and cardiac chamber enlargement supplementary to TEE [64]. Limitations include radiation exposure, costs, and the need for contrast agents [46,48]. A CT-A was performed in seven 7 of 47 tabulated cases (14.9%).

### 3.6. Differential Diagnoses to Be Considered

In the diagnosis of the Gerbode defect, the high-velocity LV-RA jet may be misdiagnosed as TV regurgitation with pulmonary arterial hypertension, Valsalva aneurysm rupture, mitral regurgitation, paravalvular leak, VSDs, or other types of congenital intracardiac shunts [65].

An indirect Gerbode defect is often misinterpreted as tricuspid regurgitation associated with pulmonary arterial hypertension because the LV-RA shunt of the Gerbode defect is typically masked behind an aneurysmal transformation of the septal leaflet of the tricuspid valve plus the LV-RA shunt direction parallels that of the TV regurgitation [60]. Both shunt blood to the RA during ventricular systole, but differ in the following aspects. Mahajan et al. reported that the finding of two separate systolic, bidirectional jets can be used to differentiate an indirect Gerbode defect, presenting with unidirectional shunt flow, from an Eisenmenger VSD with TV regurgitation [32,39]. The origin of the high-velocity jet in the Gerbode defect from the membranous portion of the interventricular septum differs from that of a TV regurgitation originating from the valve [1]. On color and continuous wave Doppler, the LV-RA shunt of the Gerbode defect presents with a higher trans-shunt pressure gradient resulting in a higher jet velocity than TV regurgitation. In addition, in a TV regurgitation, there is no step-up of oxygen saturation from the superior vena cava to the RA during cardiac catheterization, as is typical of a Gerbode defect. Cardiac MRI can be used to visualize the intracardiac high-velocity shunt of the Gerbode defect, impinging the anterior tricuspid leaflet during systole and reflecting back into the RA [5]. If this systolic shunt is misdiagnosed as TV regurgitation, pulmonary arterial hypertension will be incorrectly diagnosed. The cardiac catheterization finding of normal diastolic pulmonary arterial pressure, absence of RV hypertrophy, and lack of septal flattening allows us to exclude true pulmonary arterial hypertension and to recognize a right atrial jet of the Gerbode defect [1,7,25,37,42].

A Gerbode defect may be confused with a ruptured sinus of Valsalva aneurysm. If affecting the non-coronary sinus, it typically extends into the right atrium. Differences in the timing of the shunt flow may help to distinguish between these two lesions. The Gerbode defect typically produces a left-to-right shunt during systole, whereas in ruptured sinus Valsalva aneurysms, additionally, diastolic shunting caused by the diastolic gradient between the aorta and the RA is perceived [1,7,44,52].

### 3.7. Choice of Treatment Option

Due to the rare occurrence and difficulty in diagnosing the Gerbode defect, there is no medical evidence or official guidance regarding an optimized therapeutic strategy [37,66]; instead, this is based, in particular, on case reports or case series [12]. Possible treatment options include conservative monitoring, cardiac surgery, or interventional device closure [37]. The selection of individual therapy should be based on the presence of chamber enlargement and the severity of symptoms, which depends on factors such as the size, location, and magnitude of the shunt, flow volume, and concomitant cardiac abnormalities [1,11,67]. Consequently, asymptomatic patients presenting with an insignificant small LV-RA shunt, without circulatory overload, may be managed by conservative follow-up without accessing surgical closure [1]. This assumption can be supported by the observation of spontaneous closure of the Gerbode defect by the formation of a tricuspid valve leaflet aneurysm covering the defect. However, this occurs very rarely, particularly in congenital cases of the Gerbode defect (7%) [5,12,24]. However, a significant LV-RA shunt, presenting with cardiac chamber enlargement, progression, and/or suspected TV damage, requires therapeutic correction [31]. This recommendation is based on the following reasons: firstly, untreated significant shunts increase the risk of progressive congestive heart failure, infective endocarditis, and pulmonary hypertension in long-term follow-up, and secondly, a surgical closure is reported to be a safe procedure with a successful outcome, avoiding progression of TV regurgitation, clinical deterioration, and occurrence of infective endocarditis. Percutaneous catheter closure techniques had been increasingly used in acquired cases of the Gerbode defect as well as in high-risk surgical patients, but meanwhile, they are increasingly being applied [37,57,68]. Nevertheless, in the literature there even exists the view that every shunt should be corrected, regardless of its hemodynamic relevance and the severity of symptoms [7,9,12,23].

### 3.8. Surgical Closure

Surgical closure represents the conventional and well-established method of treatment for all types of Gerbode defects, presenting with positive outcomes as well as low morbidity and mortality, but requires open-heart surgery with cardiopulmonary bypass and cardioplegic diastolic arrest [31,40,69,70]. In the case of a smaller defect, a direct suture repair may be possible, whereas extended defects require the insertion of a pericardial or synthetic patch on the right atrial side in order to reduce the risk of recurrence and to prevent complications such as atrioventricular block [12,53,67]. Other surgical techniques include patch closure by suturing from the ventricular side, passing the tricuspid valve leaflets, or Dacron patch closure with reimplantation of the septal leaflet onto the patch [7].

Kelle et al. reported on a series of six patients suffering from congenital Gerbode defect who underwent surgical repair. All of these presented as being symptomatic along with an enlargement of the RA. Regardless of defect size, all patients were treated using the patch technique. This procedure was shown to be safe with no mortality and without complications such as arrhythmias, residual shunts, recurrence, and tricuspid regurgitation. Thus, they recommend the surgical approach for the treatment of the congenital Gerbode defect [51].

### 3.9. Device Closure

As an alternative therapy option to surgical closure, percutaneous transcatheter closure using septal occluders is gaining more importance and utilization. Sternotomy and bypass are thereby avoided. A variety of different devices are used, including Amplatzer duct occluder (ADO) I, ADO II, Amplatzer muscular ventricular septal defect occluder, Cera ductal occluder, and Nit-occluder Le VSD Coil [12,37,53,71]. The aforementioned devices have been developed for the treatment of certain other cardiac defects such as atrial septal defects, ventricular septal defects, persistent ductus arteriosus, and others. Therefore, due to the lack of devices specialized for the treatment of Gerbode defects, these devices are used off label [37]. The choice of device depends on the size of the defect, proximity to the conduction system, and the surgeon’s preference. The following aspects should be considered: (1) Proximity to atrioventricular conduction bundles: Monitoring of cardiac rhythm for early detection of heart block; the muscular ventricular septal defect occluder, presenting with its short central stent and higher radial force, tends to compress the perinodal tissue and favors the occurrence of an AV block. (2) Proximity to the septal leaflet of the tricuspid valve: using intraoperative TEE, the new onset of tricuspid regurgitation should be investigated; the use of an ADO provides an appropriate fit due to the absence of a right-sided disc and the left ventricular location of the retention plate. (3) Proximity to the aortic valve: post-device closure monitoring of the left ventricular outflow tract by means of intraoperative TEE. (4) Proximity to coronary sinus, verifying non-affection by TEE and electrocardiogram [59,72].

Due to these aspects, device closure was considered to be problematic in infants and children. Recently, the ADO II has gained increasing importance in pediatric cardiac surgery and is thought to be the most appropriate, safe, and effective device, reducing the complication rate [45,73]. Advocacy for the use of the ADO II is supported by a series of 12 successful cases of retrograde closure of various types of congenital Gerbode defects using the ADO II reported by Vijayalakshmi et al. Patient ages ranged from ten months to 16 years. None of the patients showed postoperative aortic or tricuspid regurgitation, or a residual shunt, and only one patient developed a transient heart block, which resolved after temporary pacing. This thus confirms that device occlusion is not limited to the adult population [45]. Its advantages include low radial force, low profile, and soft retention discs [7,12,74]. Due to the central waist of the ADO II, it adapts to the defect, while the soft, freely movable retention discs on both sides do not interfere with the nearby valves and the conducting system [57]. The size of the device is chosen to be 1–2 mm more than the maximum LV entry defect diameter measured by TEE at the end of diastole and by an LV angiogram, in order to avoid over- and under-sizing [24,45]. In addition, the procedure time is shortened and the device costs are reduced by one-third. Available device sizes, however, restrict the use of the ADO II to defects with a maximum diameter of 6 mm [74]. Haddad et al. reported on the KONAR Multifunctional occluder, which represents a newly off-label approach with expanded size availability but decreased flexibility of the left ventricular retention disc [57]. However, prospective large studies are needed to evaluate the safety and applicability of the ADO II in children with congenital Gerbode defects.

Data extraction from our case series analysis revealed information regarding the therapeutic approach in 37 of 47 patients (78.7%). Most of the patients presenting with congenital Gerbode defects underwent conventional surgical closure, i.e., 22 patients (59.5%). Device closure was used in ten cases (27.0%), with the most commonly used device being the ADO (five cases; 50.0%). Further devices used included the Amplatzer ventricular septal defect occluder (two cases; 20.0%) as well as the Cera duct occluder, Nit-occluder Le VSD coil, and multifunctional occluder in one case each (10.0% respectively). Five cases (13.5%) were followed up with a conservative therapeutic strategy.

The choice of treatment for Gerbode defects appears not to correlate with the subtype of defect identified. Surgical closure is the predominant treatment across all three types (type I, 58.3%; type II, 60.0%; type III, 60.0%), indicating its effectiveness in addressing the anatomical complexities of these defects. Type III defects show a more balanced distribution between surgical closure (60.0%) and conservative management (40.0%), possibly reflecting a perceived clinical stability in some cases. Overall, no association can be found between the choice of treatment option and the type of Gerbode defect.

In all patients, a positive outcome with survival and clinical improvement, irrespective of the therapeutic strategy used, is described.

### 3.10. Complications

Surgical as well as interventional therapy of the Gerbode defect might be challenging due to its anatomic proximity to atrioventricular conduction bundles, aorta, and tricuspid and mitral valves. Potential sequelae of surgical closure include infection, postoperative bleeding, valve injury, pulmonary hypertension with poor cardiac output, atrial tachycardia, arrhythmias, and complete heart block. Improved surgical techniques and intraoperative real-time monitoring by TTE have increased the rate of success and decreased the rate of complications [46,69].

During device closure, residual shunts, new or increased tricuspid regurgitation, dislocation of the device, and intra- and post-procedural atrioventricular block frequently occur [31,40,50]. A transient or permanent complete heart block is a frequently observed complication of transcatheter closure, which is described to occur immediately after device implantation. The occurrence of a complete heart block is more frequently observed in the closure of indirect Gerbode defects compared to direct defects [57]. The use of innovative soft devices, like ADO II and Multifunctional occluder devices, with a reduced radial force, decreased the rate of complications such as complete heart blocks [67]. In addition, hemolysis has been reported after transcatheter occlusion. Hemolysis may be transient, due to device thrombosis and endothelialization, or persistent, leading to acute kidney injury and ultimately to surgical removal of the device [75]. The supportive wire frame and looser mesh fabric weave, leading to red blood cell shearing, are thought to be the cause of the more frequent occurrence of hemolysis using Amplatzer occluders [75].

If left untreated, the left-to-right shunt can result in early and progressive heart failure with hemodynamic deterioration, exercise intolerance, cardiac chamber enlargement, and valve destruction along with an annular abscess, usually within six months [5,50,69]. The study by Chou et al. indicated an association between an LV-RA shunt presenting with right atrial enlargement and the presence of atrial fibrillation. Structural and electrophysiological remodeling is thought to be the underlying mechanism. A higher incidence of atrial fibrillation in patients with a significant Gerbode defect increases the risk of stroke, pulmonary embolism, and heart failure, leading to a subsequent increase in cardiac mortality [76]. In addition, untreated Gerbode defects are associated with an increased incidence of infective endocarditis. Therefore patients with a known septal defect should be included as high-risk patients in the guidelines for receiving endocarditis prophylaxis [49].

### 3.11. Analysis of Included Case Reports of Congenital Gerbode Defect Based on Age Groups

Analysis of the included case reports (see Table 2) of congenital Gerbode defects reveals a diverse distribution across age cohorts for subgroup analysis. These cohorts were categorized as follows: newborns or infants (0 to 2 years), children (3 to 16 years), young adults (17 to 30 years), and middle to old-aged adults (over 30 years).

The age at initial diagnosis of a congenital Gerbode defect, as depicted in the analyzed case reports, exhibits a wide range, spanning from one month to 76 years, with a median age of 17 years.

A comparison between the age at initial diagnosis and the presence of symptoms provides insights into the natural progression of the disease across different age groups. Notably, all cases within the newborn and infant age group presented with symptoms, indicating early onset and the acute nature of presentation in this age group. In contrast, among children, only 55.6% of cases were symptomatic, suggesting a significant proportion of asymptomatic presentations in this cohort. Conversely, in the middle- and old-aged adult group, the symptomatic percentage surged to 87.5%, indicative of a progressive nature of the disease with advancing age.

Furthermore, treatment modalities varied according to age group, reflecting potentially distinct disease characteristics and therapeutic approaches tailored to age-specific characteristics. Surgical intervention predominated in newborn and infant cases (88.9%), highlighting the importance of early intervention in this vulnerable population. Device closure emerged as a common therapeutic approach in children (46.2%) and young adult age groups (50.0%), likely owing to considerations of minimally invasive options and developmental stage. In contrast, conservative management was more frequently observed in middle- and old-aged adults (36.4%), possibly reflecting a balance between risk and benefit in this population. Nevertheless, surgical therapy remained the predominant treatment modality across all age groups, underscoring its significance in managing congenital Gerbode defects.

These observations suggest age-dependent variations in clinical presentation, disease progression, and treatment strategies, underscoring the need for tailored approaches to optimize patient outcomes across the lifespan.

## 4. Discussion

By conducting a systematic review of the literature, this study aimed to consolidate existing evidence, shed light on the clinical implications of the congenital Gerbode defect, and emphasize the importance of early diagnosis and intervention to enhance patients’ quality of life. By providing an overview of its various clinical presentations, appropriate diagnostic modalities, and therapeutic strategies, this review aims to increase pediatricians’ awareness of the clinical picture of the Gerbode defect in order to prevent the development of any long-term complications due to delayed initiation of therapy.

The Gerbode defect presents a major diagnostic challenge due to the following aspects. It is a rare congenital cardiac anomaly with a very low reported incidence of less than 1% of all congenital cardiac defects [77]. Its etiology, pathogenesis, and embryology are not fully understood; thus, no screening approach based on the presence of underlying causal risk factors exists. In addition, the Gerbode defect manifests with a large variety of symptoms depending on the size and severity of the shunt. Clinical presentation, even in our case series, ranges from asymptomatic over dyspnea and palpitations to signs of heart failure [66]. The 25% of asymptomatic patients reported by Yuan [5] is consistent with the evaluated 20.51% reported in our case review. The most common symptoms included dyspnea, palpitations, shortness of breath, fatigue, and growth restriction, which is consistent with symptoms reported by other authors [1,12]. The similarity of several clinical manifestations of the Gerbode defect with more common clinical pictures, such as TR, pulmonary arterial hypertension, or other intracardiac shunts, may result in a misdiagnosis and a delay of a correct diagnosis. Thus, the acquisition of symptomatology is not sufficient for a definitive diagnosis of a Gerbode defect, and further investigative techniques, like echocardiography, MRI, CT-A, and catheterization, are required for an accurate assessment of the shunt [1]. Nevertheless, a detailed patient history, as well as a physical examination, represent a fundamental part of any diagnostic workup and provide a valuable guide for achieving an early diagnosis and planning of interventions to prevent the progression and complications of the disease [39]. Indicative of the presence of a Gerbode defect is a loud, harsh, grade III-IV holosystolic murmur occurring along the left sternal border in the fourth or fifth intercostal spaces in 92.31% of documented cases suffering from a congenital Gerbode defect. Additionally, the analysis of case reports on congenital Gerbode defects reveals a varied distribution of symptoms across age groups, highlighting distinct clinical presentations. The age at first diagnosis ranges from infancy to adulthood, with newborns usually presenting with symptoms, whereas children and young adults often present as asymptomatic. The proportion of symptomatic patients tends to increase with older age, indicating a progressive nature of the disease.

For a more accurate detection and evaluation of the Gerbode defect, a multimodal diagnostic approach is recommended by many authors [5,37,46,78]. TTE, which is considered to be the diagnostic modality of choice [1], is limited in its accuracy to detect the shunt, facilitating an accurate diagnosis in only 62% of cases, and frequently leading to misdiagnosis [37,44,66]. Despite the shortcomings of TTE, including limited diagnostic accuracy and the high rate of misdiagnoses and missed diagnoses, it provides the most appropriate noninvasive modality for diagnosing Gerbode defects in newborns because generating a clear image is easier in children and adolescents than in adults [40]. It should be performed promptly by a pediatric expert in cases of clinical suspicion to prevent a delay in diagnosis and to improve the patient’s outcome. Various echocardiographic clues exist, such as persistent diastolic flow due to a high LV-RA Doppler gradient, normal pulmonary artery pressure, and the absence of ventricular septal flattening and RV hypertrophy [33]. Fetal echocardiography can be used to diagnose an atrioventricular septal defect beginning at the twelfth week of gestation in specialized centers. Rojas et al. reported that specific screening programs, which included distinct protocols and adequate training of sonographers, can increase the detection rate from 27% to as high as 93% [79].

Complementary diagnostic modalities, such as 3D TEE or cardiac MRI, may allow a more precise definition of the anatomy and hemodynamics of the shunt, depending on the age of the patient, in order to optimize the therapeutic strategy on an individual basis [5,80,81]. Chest X-ray, CT-A, and cardiac catheterization should be used only secondarily in neonates with suspected congenital Gerbode defects because of their radiation exposure and invasiveness, respectively [8].

After a careful review of the literature, we can establish the following key points in the diagnostic workup, as applied to infants, of congenital Gerbode defects. A detailed medical history regarding clinical symptoms such as dyspnea, palpitations, shortness of breath, fever, and growth retardation should be performed. In the presence of a harsh, loud, pansystolic murmur at the left sternal border, noninvasive diagnostics such as an electrocardiogram and a TTE, optimally with 3D enhancement, should be performed with attention paid to the clues of Silbiger et al. [52]. In case this is not sufficient for an accurate evaluation of the shunt, a complementary MRI or TEE may be performed.

An accurate diagnosis has a major impact on clinical decision-making when choosing a therapeutic strategy [82]. This should be made individually depending on the symptomatology, the age, and anatomy of the shunt. Currently, surgical closure offers the most secure option for the treatment of infants and newborns due to the high evidence for a successful outcome and the low risk of complications. However, further development of the device closure technique could increase its importance due to its lower invasiveness and recovery time. In terms of therapy of the congenital Gerbode defect, this procedure is still in its infancy. Conservative treatment is often chosen in the older patient group due to risk–benefit considerations. Nevertheless, all patients with a congenital Gerbode defect tabulated by us resulted in a positive outcome with survival and clinical improvement, irrespective of the therapeutic strategy used.

However, it should be noted that the clinical picture of the Gerbode defect represents an extremely rare cardiac anomaly in which a spontaneous closure without hemodynamic relevance and clinical significance is even possible. Consequently, many cases will remain undetected, resulting in a high undiagnosed rate. Due to the wide spectrum of clinical significance, from asymptomatic up to heart failure, overdiagnosis and overtreatment should be avoided in order to reduce the burden on the health care system as well as on the patients themselves.

This systematic literature review has certain limitations. The research field around the congenital Gerbode defect is rather small and adynamic due to the low incidence. Due to the small number of cases, little literature is available that concretely addresses the congenital form. Most of the evidence is based on case reports with little original or basic research studies existing. Further investigation is needed to provide evidence-based guidance on the suitability of diagnostic modalities and treatment options, particularly in neonates. Moreover, there is a gap in the literature regarding the pathogenesis, resulting in a lack of causal approaches in prevention, screening, and therapy. As it represents a major diagnostic challenge, pediatricians need to be sensitized regarding the importance of the clinical picture of the Gerbode defect in order to reduce complications caused by deficiency of therapy. Prospective studies on prenatal diagnosis of the Gerbode defect are lacking, but these studies would be of elementary importance for prompt intervention.

Our motivation for conducting this study stems from our commitment to improving prenatal diagnostic techniques and ensuring that early and accurate detection of such defects can be achieved. By enhancing our understanding and awareness of Gerbode defects, we aim to contribute to the early diagnosis and intervention strategies, ultimately improving outcomes for affected neonates and supporting the multidisciplinary approach required in managing these complex cases. As gynecologists, we are deeply involved in the prenatal diagnosis of congenital heart malformations, but we recognize that our perspective may not encompass the intricacies and nuances of pediatric cardiology as extensively as those of specialists in the field. However, in Germany, the diagnosis and management of fetal and congenital heart malformations, including Gerbode defects, is predominantly incorporated within the domain of prenatal physicians. This is not solely the domain of pediatric cardiologists. Given the significant role that prenatal diagnosis plays in identifying congenital anomalies, Gerbode defects are also highly relevant to the fields of gynecology and obstetrics.

## 5. Conclusions

Although the Gerbode defect is a rare congenital cardiac anomaly, it has a significant impact on patients’ quality of life. This review highlights the importance of an accurate diagnosis for early initiation of therapy to avoid limiting complications. Large literature gaps were found regarding specific evidence of the congenital form of the Gerbode defect. Therefore, further studies are needed to optimize diagnostic approaches and management strategies in neonates and to improve patient outcomes and quality of life.

## Figures and Tables

**Table 1:** Eligibility criteria. Eligibility criteria were defined according to the PICOM scheme and served as a decision-making basis in the study selection process.

Population	patients suffering from congenital Gerbode defect
Phenomenon of Interest	general data, pathophysiology, diagnostics, and treatment
Context	a systematic review of the literature
Methodology	a systematic literature search of case reports, expert reviews, and original studies

**Table 2:** Cases of congenital Gerbode defect and their characteristics. All cases of congenital Gerbode defect described in the literature including their sociodemographic data, symptoms, diagnostic findings, therapeutic strategies, and outcome are compiled.

Author(s)	Gender	Age at Diagnosis	Symptoms	Diagnostic Tool								Type of GD	Treatment	Outcome
				Auscultation	ECG	2D/3D TTE	2D/3D TEE	CXR	CC	MRI	CTA			
Acar et al. [4]	M	14 y	-	+	n.a.	+/+	n.a.	n.a.	n.a.	n.a.	n.a.	II	Conservative	n.a.
Agoston et al. [16]	M	76 y	Chest pain, dyspnea	+	n.a.	+/+	n.a.	n.a.	n.a.	n.a.	n.a.	II	Conservative	Improved
Blanco et al. [17]	M	27 y	Shortness of breath	n.a.	-	+/n.a.	+/n.a.	n.a.	n.a.	+	n.a.	III	Surgery	n.a.
Borkar et al. [10]	M	31 y	-	-	n.a.	+/n.a.	n.a.	n.a.	n.a.	n.a.	n.a.	II	n.a.	n.a.
Borkar et al. [10]	F	27 y	-	+	n.a.	+/n.a.	n.a.	n.a.	n.a.	n.a.	n.a.	I	n.a.	n.a.
Borkar et al. [10]	F	55 y	Fever, palpitations	-	n.a.	+/n.a.	n.a.	n.a.	n.a.	n.a.	n.a.	I	n.a.	n.a.
Colomba et al. [18]	F	48 y	Dyspnea	+	n.a.	+/n.a.	n.a.	n.a.	n.a.	n.a.	n.a.	II	n.a.	n.a.
Demirkol et al. [19]	M	21 y	Shortness of breath	+	+	+/+	+/+	n.a.	n.a.	+	n.a.	I	n.a.	n.a.
Desai et al. [20]	F	37 y	n.a.	n.a.	n.a.	+/n.a.	+/n.a.	n.a.	+	+	n.a.	II	Surgery	Survived
Du et al. [8]	F	2 y	Sweating, poor appetite	+	n.a.	+/n.a.	n.a.	n.a.	n.a.	n.a.	n.a.	I	Surgery	Survived
Erdöl et al. [21]	M	20 y	-	-	-	+/n.a.	n.a.	-	n.a.	n.a.	n.a.	I	n.a.	n.a.
Eroglu et al. [22]	M	63 y	Dyspnea	+	n.a.	+/n.a.	n.a.	n.a.	+	n.a.	n.a.	I	n.a.	n.a.
Flores et al. [23]	M	2 m	n.a.	+	+	+/n.a.	n.a.	+	n.a.	n.a.	+	II	Surgery	Survived
Ganesan et al. [24]	F	26 y	Shortness of breath, fatigue, dizziness on mild exertion	+	n.a.	+/n.a.	+/n.a.	n.a.	n.a.	n.a.	n.a.	II	Cera duct occluder	Survived
Ganju et al. [25]	F	2y	Failure to thrive	+	n.a.	+/n.a.	n.a.	+	n.a.	n.a.	n.a.	III	Surgery	n.a.
Gerbode et al. [26]	M	15 y	Fatigue, dyspnea	+	+	n.a.	n.a.	+	+	n.a.	n.a.	II	Surgery	Survived
Gerbode et al. [26]	F	18 m	Failure to thrive, fatigue, bilateral clubbed feet	+	+	n.a.	n.a.	+	+	n.a.	n.a.	I	Surgery	Survived
Gerbode et al. [26]	M	5 y	Dyspnea, palpitations	+	+	n.a.	n.a.	+	+	n.a.	n.a.	III	Surgery	Survived
Gerbode et al. [26]	M	42 y	Dyspnea, palpitations	+	+	n.a.	n.a.	+	+	n.a.	n.a.	I	Surgery	Survived
Gerbode et al. [26]	F	17 y	Fatigue, palpitations	+	+	n.a.	n.a.	+	+	n.a.	n.a.	II	Surgery	Survived
Gościniak et al. [27]	M	52 y	Headache	+	+	+/n.a.	+/n.a.	+	n.a.	n.a.	n.a.	III	n.a.	n.a.
Gunaydin et al. [28]	M	22 y	Dyspnea	+	+	+/+	n.a.	n.a.	n.a.	n.a.	n.a.	I	ADO	Survived
Gur et al. [29]	F	14 y	Dyspnea, chest pain	+	+	+/n.a.	n.a.	n.a.	n.a.	n.a.	n.a.	II	Surgery	Survived
Haponiuk et al. [30]	M	8 m	n.a.	n.a.	+	+/n.a.	n.a.	+	n.a.	n.a.	n.a.	I	Surgery	Survived
Kerst et al. [31]	F	12 y	n.a.	n.a.	-	+/n.a.	n.a.	n.a.	n.a.	n.a.	+	II	AMVSDO	Survived
Kerst et al. [31]	M	9 y	n.a.	n.a.	-	+/n.a.	n.a.	n.a.	n.a.	n.a.	+	I	ADO II	Survived
Kerst et al. [31]	M	6 y	n.a.	n.a.	-	+/n.a.	n.a.	n.a.	n.a.	n.a.	+	II	ADO II	Survived
Kerst et al. [31]	M	6 y	n.a.	n.a.	-	+/n.a.	n.a.	n.a.	n.a.	n.a.	+	II	AMVSDO	Survived
Mahajan et al. [32]	F	35 y	Dyspnea, palpitations	+	+	+/n.a.	n.a.	n.a.	+	n.a.	+	II	Surgery	Survived
Martino et al. [33]	F	1 m	Intrauterine growth restriction, respiratory distress, fatigue	+	-	+/n.a.	n.a.	n.a.	n.a.	n.a.	n.a.	II	Surgery	Survived
Mateescu et al. [6]	F	55 y	Dyspnea	+	-	+/n.a.	+/n.a.	+	+	n.a.	n.a.	I	Surgery	Survived
Mehdi et al. [9]	M	2 m	n.a.	n.a.	n.a.	+/n.a.	n.a.	n.a.	+	+	+	I	n.a.	n.a.
Müller et al. [34]	M	55 y	-	+	-	+/n.a.	n.a.	n.a.	+	+	n.a.	I	Surgery	Survived
Otaigbe and Orubide [35]	F	5 m	Failure to thrive, respiratory distress	+	n.a.	+/n.a.	n.a.	+	n.a.	n.a.	n.a.	II	Surgery	Survived
Panduranga and Mukhaini [36]	M	13 y	-	+	+	+/n.a.	+/n.a.	+	n.a.	n.a.	n.a.	II	Surgery	Survived
Phan et al. [37]	M	31 y	Dyspnea	+	-	+/n.a.	n.a.	-	+	n.a.	n.a.	II	Nit-Occlud^®^ Lê VSD coil	Survived
Quien et al. [38]	M	38 y	Shortness of breath, dyspnea	+	+	+/n.a.	n.a.	+	+	n.a.	n.a.	I	conservative	Survived
Rani et al. [39]	W	55 y	Palpitation, dyspnea	+	-	+/n.a.	+/n.a.	-	n.a.	n.a.	n.a.	II	Surgery	Survived
Roy et al. [40]	W	8 y	Failure to thrive	+	-	+/n.a.	n.a.	n.a.	n.a.	n.a.	n.a.	III	ADO	Survived
Roy et al. [40]	M	11 m	Chest infections, failure to thrive	+	-	+/n.a.	n.a.	n.a.	n.a.	n.a.	n.a.	III	Multifunctional occluder	Survived
Roy et al. [40]	M	5 y	-	+	-	+/n.a.	n.a.	n.a.	n.a.	n.a.	n.a.	I	ADO II	Survived
Singh et al. [3]	F	2 m	Fever, respiratory distress	+	n.a.	+/n.a.	n.a.	+	n.a.	n.a.	n.a.	II	Surgery	Survived
Tacoy et al. [41]	M	48 y	Palpitations, shortness of breath	+	-	+/n.a.	+/n.a.	n.a.	n.a.	+	n.a.	I	Conservative	n.a.
Tehrani and Movahed [42]	F	49 y	Fatigue, palpitations	+	-	+/n.a.	n.a.	n.a.	n.a.	n.a.	n.a.	II	n.a.	n.a.
Tidake et al. [43]	M	8 y	-	+	+	+/n.a.	n.a.	+	n.a.	n.a.	n.a.	I	Surgery	Survived
Tidake et al. [43]	M	10 y	Shortness of breath, fever, cough	+	n.a.	+/n.a.	n.a.	+	n.a.	n.a.	n.a.	II	Surgery	Survived
Uddin et al. [44]	F	50 y	Dyspnea, chest pain, disturbed sleep	+	+	+/n.a.	n.a.	+	n.a.	n.a.	n.a.	II	Conservative	Survived

F—female; M—male; y—years; m—months; n.a.—not available; + With pathological findings; - Without pathological findings; ECG—electrocardiogram; TTE—transthoracic echocardiography; TEE—transesophageal echocardiography; CXR—chest X-ray; CC—cardiac catheterization; MRI—cardiac Magnetic resonance imaging; CTA—computed tomographic angiography; GD—Gerbode defect; ADO—Amplatzer duct occluder; AMVSDO—Amplatzer muscular ventricular septal defect occluder.

## Data Availability

Data available on request from the authors.

## Abbreviations

LV: left ventricle, RA: right atrium, TV: tricuspid valve, RV: right ventricle, VSD: ventricular septal defect, TTE: transthoracic echocardiography, TEE: transesophageal echocardiography, MRI: magnet resonance imaging, CT-A: computed tomography angiography, ADO: Amplatzer duct occlude.
